# Differences in the Clinical Characteristics of Early- and Late-Onset Necrotizing Enterocolitis in Full-Term Infants: A Retrospective Case-Control Study

**DOI:** 10.1038/srep43042

**Published:** 2017-02-17

**Authors:** Qiu-Yu Li, Yao An, Li Liu, Xue-Qiu Wang, Shi Chen, Zheng-Li Wang, Lu-Quan Li

**Affiliations:** 1 Neonatal Diagnosis and Treatment Center, Children’s Hospital of Chongqing Medical University; Ministry of Education Key Laboratory of Child Development and Disorders; China International Science and Technology Cooperation base of Child Development and Critical Disorders; Key Laboratory of Pediatrics in Chongqing. Chongqing, 400014, P.R. China

## Abstract

Information regarding the influence of age at onset on prognosis in full-term infants with necrotizing enterocolitis (NEC) is limited, and identifying differences between the clinical characteristics of early-onset NEC (EO-NEC) and late-onset NEC (LO-NEC) may be helpful in the determination of effective management strategies. In the present study, the medical records of 253 full-term infants with NEC were reviewed, and the clinical characteristics of the EO-NEC group (n = 150) and the LO-NEC group (n = 103) were compared. Infants in the EO-NEC group were characterized by increased gestational age and higher rates of stage III NEC and peritonitis when compared with LO-NEC infants (*P * < 0.05). Mortality was significantly associated with stage III NEC, peritonitis, sepsis, respiratory failure and shock in univariate analysis (*P* < 0.05). In logistic regression analysis, peritonitis and renal failure were identified as independent risk factors for mortality in infants with EO-NEC, and peritonitis and respiratory failure were significant predictors of mortality in neonates with LO-NEC. Our finding indicated that the characteristics of the severe medical conditions identified in infants with EO-NEC were distinct from those observed in infants with LO-NEC. Peritonitis and kidney failure and peritonitis and respiratory failure were identified as risk factors for mortality in EO-NEC and LO-NEC infants, respectively.

Necrotizing enterocolitis (NEC) is a common and devastating inflammatory gastrointestinal disease that has been found to occur at a rate of 1–5 infants per 1000 live births^1^. The mortality rates associated with NEC have been found to range from 20 to 30%^1^,^2^, and despite the rapid evolution of medical technology over the past three decades, mortality rates among infants requiring surgery may be even higher^3^,^4^,^5^. Although the pathogenesis of NEC is multifactorial, and the exact etiology of the disease remains unclear, gastrointestinal tract and immune system immaturity have been identified as one of the most important causes of this condition^1^. Due to differences in intestinal and immune system maturity between preterm and term infants, the clinical characteristics of NEC may be fundamentally different in these two groups. Previous studies have indicated that in full-term infants, NEC is characterized by a younger age at onset^6^,^7^,^8^ and greater rates of cardiac disease^6^, pneumatosis intestinalis and intrahepatic venous gas, lower abdominal distention, and ileus^8^ when compared with preterm infants; additionally, NEC is most frequently identified within 7 days of birth in this patient population^6^,^9^,^10^,^11^. Short *et al*.^9^ found that among full-term infants, the development of NEC after 7 days of life was associated with increased mortality. This finding suggests that age at NEC onset may affect prognosis in full-term infants. Thus, the identification of intrinsic risk factors for mortality in different segments of this patient population may inform the implementation of effective management strategies. However, the results of that pilot study (n = 39)^9^ may not provide a comprehensive profile of NEC in term infants, and studies including larger samples of patients should be conducted. The aim of this study was to clarify the influence of age at onset and internal risk factors on mortality in full-term infants with NEC by comparing the characteristics of this condition among infants in whom NEC was identified within 7 days of life and infants in whom NEC was identified 7 or more days post-birth.

## Methods

### Data collection

The medical records of all full-term infants (gestation age ≥37 weeks) with NEC (Bell’s stage ≥II) who had been admitted to the Children’s Hospital of Chongqing Medical University within 28 days of life between Feb 1996 and Dec 2015 were reviewed. NEC was staged according to the criteria originally proposed by Bell *et al*.^12^ and subsequently modified by Walsh and Kliegman^13^. NEC was diagnosed based on the presence of one or more of the parenthesized clinical signs (bilious gastric aspirate or emesis, abdominal distention, and occult and/or gross blood in stool (no fissures)) and the presence of at least one of the following three radiographic or sonographic findings: (1) pneumatosis intestinalis, (2) portal vein gas and/or (3) pneumoperitoneum. If gastrointestinal perforation was identified based on visual inspection of the bowel at the time of surgery or post-mortem examination, the individual was not categorized as having NEC and, therefore, excluded from this study. Patients with congenital gastrointestinal malformations, with incomplete medical information or on whom surgical intervention after perforation was not performed were also excluded. All infants received similar interventional protocols including broad-spectrum antibiotic therapy, intensive care, cessation of enteral feeding, nasogastric suction and parenteral nutrition. Cardiorespiratory support, transfusion of blood or blood products or surgical intervention were also provided when necessary.

Following patient identification, all information pertaining to NEC that had been recorded by physicians or nurses during patient hospitalization, including demographic, laboratory examinations, abdominal radiographic and sonographic, surgical and outcomes data, were reviewed and extracted. Age at NEC onset was determined based on the age of the patient on the day on which at least one of the following signs or symptoms was identified: pre-feeding gastric residuals, abdominal distention, emesis, bloody stool and/or diarrhea. Age at diagnosis was determined based on the age of the patient on the day on which abdominal X-ray or sonographic findings indicated that the patient fulfilled Bell’s criteria (having pneumatosis intestinalis, portal venous gas or both). Similar to a previous study^9^, early-onset NEC (EO-NEC) was defined as NEC diagnosed ≤ 7 days post-birth, and late-onset NEC (LO-NEC) onset was defined as NEC diagnosed more than 7 days post-birth. This retrospective, observational study was conducted in accordance with institutional guidelines in a level III neonatal intensive care unit. This retrospective study was approved by the Ethics Committee of the Children’s Hospital of Chongqing Medical University (approval No. 2016–19), and use of the database housing the evaluated data was permitted by the ethics committees of the Children’s Hospital of Chongqing Medical University. The data were collected, reviewed, de-identified, and anonymously analyzed by the authors, and the Ethics Committee waived the requirement for informed consent because of the anonymized nature of the data and scientific purpose of the study.

### Statistical analysis

All data were analyzed using SPSS 13.0 (SPSS Inc. Chicago, IL). Continuous variables were tested for normality using the Kolmogorov-Smirnov test. Normally distribution data are described as the means ± standard deviations (the mean ± S.D. of the variable) and were analyzed using Student’s *t* test. Skewed data are described as medians and interquartile ranges (IQRs) and were analyzed using the Mann-Whitney *U* test. Categorical data were analyzed using the Chi-square test or Fisher’s exact test. Multivariate regression analyses were performed to identify independent risk factors for mortality. Statistical significance was established at *P* < 0.05.

## Results

### Clinical characteristics of NEC in full-term infants

During the study period, 70,326 full-term infants were admitted to the Children’s Hospital of Chongqing Medical University, 291 (0.41%) of whom developed NEC. A total of 253 NEC infants met the inclusion criteria, and 38 cases were excluded due to intestinal malformation (n = 10), incomplete information (n = 17) and an age at onset >28 days (n = 11). The demographic characteristics of the infants are shown in Table 1. Sixty percent of the infants (n = 154) were male, and the average gestational age (GA) and birth weight of the patients were 39 weeks and 3110 g, respectively. Intrauterine growth retardation was identified in 22% (n = 56) of the patients, and formula feeding prior to NEC onset was identified in more than 70% (n = 179) of the included infants. The median onset age was 6 days, and medium age at diagnosis was 8 days. Fifty-four of the 253 included infants developed stage III NEC, and 44 of the included infants underwent surgical intervention. The overall mortality rate associated with NEC was 14.2% (36/253), and the rate of mortality among patients with stage III NEC was higher than that identified among stage II NEC patients [44.4% (24/54) vs. 6% (12/199), *χ*^*2*^ = 51.356, *P* = 0.000].

Differences identified in the baseline characteristics of patients in the LO-NEC and EO-NEC groups are described in Table 1. Infants in the EO-NEC group were characterized by increased gestational age and a higher rate of stage III NEC when compared with LO-NEC infants (*P* < 0.05). No other demographic characteristics were found to differ between two groups, and insignificant differences were identified for the gender; twin status; birthweight; feeding type; small for gestational age; and maternal diabetes, prolonged rupture of the membranes, pregnancy induced hypertension and intrahepatic cholestasis of pregnancy variables (*P* > 0.05).

The most commonly identified comorbidity was sepsis (5.1%, n = 13), followed by hypothermia (4.7%, n = 12) and hypoglycemia (4%, n = 10). Approximate 23% (n = 60) of the neonates had atrial septal defects (ASD). Other frequently observed congenital heart defects included patent ductus arteriosus (PDA, 7.1%, n = 18) and ventricular septal defects (VSD, 2.8%, n = 7). These conditions were not found to differ significantly between the early-onset group and the late-onset group (*P* > 0.05).

Major complications occurring after NEC diagnosis included sepsis (27.7%, n = 70), peritonitis (15.9%, n = 40), respiratory failure (5.9%, n = 15), shock (3.2%, n = 8), kidney failure (2.8%, n = 7), heart failure (0.8%, n = 2), multiple organ dysfunction syndrome (0.8%, n = 2), pulmonary hemorrhage and disseminated intravascular coagulation (0.4%, n = 1). Peritonitis was identified more frequently in the EO group than in the LO group (20.7% vs. 8.7%, *χ*^*2*^ = 6.528, *P* = 0.011). No significant differences in the rates of other complications, such as sepsis, respiratory failure, shock and heart failure, were identified between the EO group and the LO group (*P* > 0.05).

### Comparison of mortality

The rate of mortality did not differ significantly between the two groups [16% (24/150) vs. 11.7% (12/103), *χ*^*2*^ = 0.974, *P* = 0.331]. However, in the EO-NEC group, the rate of mortality was higher among those with stage III NEC than those with stage II NEC [43.9% (18/41) vs. 5.5% (6/109), *χ*^*2*^ = 32.684, *P* = 0.000]. A similar result was identified in the LO-NEC group, as the rate of mortality was also significantly higher in stage III than stage II NEC patients [46.2% (6/13) vs. 6.7% (6/90), *χ*^*2*^ = 13.585, *P* = 0.000].

### Risk factors associated with prognosis in NEC patients

To evaluate the risk factors for mortality among NEC patients, geographic, congenital heart disease, comorbidity (before NEC) and complication (after NEC) data were univariately compared between the survivor group and the nonsurvivor group (Table 2). No differences were identified between these two groups for the gestational age, birthweight, age at onset, small for gestational age, congenital heart disease, hypothermia, pulmonary hemorrhage and disseminated intravascular coagulation variables (*P* > 0.05). However, respiratory failure, heart failure, shock, renal failure, and sepsis after NEC were identified significantly more frequently in the nonsurvivor group relative to the survivor group (*P* < 0.05), and infants in the nonsurvivor group also more frequently required blood transfusion and plasma support that did their surviving counterparts (*P* < 0.05).

Table 3 shows the risk factors for mortality identified in the two groups. In the EO-NEC group, stage III NEC, peritonitis, sepsis, respiratory failure, kidney failure, shock and multiple organ dysfunction syndrome were significantly associated with mortality (*P* < 0.05). Meanwhile, stage III NEC, peritonitis, sepsis and respiratory failure were associated with mortality in LO-NEC infants (*P* < 0.05).

Independent risk factors for mortality were identified using multivariate logistic regression models (Table 4). Peritonitis, respiratory failure and kidney failure after NEC were significantly associated with mortality. Peritonitis and kidney failure after NEC were identified as independent risk factors for mortality in EO-NEC infants, and peritonitis and respiratory failure were identified as independent predictors of mortality in LO-NEC infants.

## Discussion

Due to only 10% of NEC occurred in full term infants, the knowledge about these patients is still not so well known. In this study, we described the clinical characteristics of NEC in full-term infants and identified differences in the clinical characteristics of early- and late-onset NEC using data from a large sample of patients. Identification of these differences might assist in the development of strategies to treat and prevent NEC in full-term infants.

In the present study, formula feeding prior to NEC onset was identified in more than 70% of infants, and the health of 22% of the patients was compromised by intrauterine growth retardation. These findings are consistent with the results of other studies^14^,^15^. The median age at NEC onset was approximately 6 days in our study, which is similar to the onset age identified in previous studies^10^,^14^. We further found that the median ages of NEC onset were 2 days in infants with EO-NEC and 14 days in neonates with LO-NEC. Whether the differences observed in onset age play a role in the various risk factors for NEC remains unknown. Further studies should be conducted to identify the risk factors associated with different ages of onset and, thereby, more effectively prevent and treat NEC in full-term infants. In full-term infants, NEC morbidity has been found to be associated with a variety of risk factors including intrauterine growth retardation, asphyxia, formula feeding, cyanotic congenital heart disease, sepsis, neonatal respiratory distress syndrome, and mechanical ventilation^16^. The rates of the majority of these risk factors was compared between the EO-NEC and LO-NEC groups in the present study, and no significant differences in any of these factors were identified between the two groups. Infants with EO-NEC were found to be greater in GA than LO-NEC infants, a finding that was consistent with the results of previous studies^8^,^17^. The reason why greater GA may be associated with earlier NEC onset remains unclear; however, we speculate that the presence of a more mature intestinal tract in term infants with greater GA may lead to earlier enteral feeding, which has been previously linked to earlier NEC onset^6^. Meanwhile, formula feeding was identified in the majority of the patients in our study, which has been reported to be an important risk factor for NEC. Thus, the combination of these risk factors might result in early NEC. However, further analysis could not be performed because all the included infants were born out-of-hospital, and we therefore did not know the exact date of enteral feeding initiation.

In our study, vaginal delivery was identified more frequently in EO-NEC than LO-NEC infants. The results of previous studies have indicated that vaginal delivery was associated with increased risk of EO-NEC^18^ in preterm infant populations. However, this finding was not verified in the study conducted by Son *et al*.^19^. The rate of intestinal bacterial colonization has been found to differ significantly between term and preterm infants^20^. Additionally, the rate of bacterial colonization has been found to differ in term infants depending on whether they were delivered vaginally or via caesarean section^21^. Thus, the findings derived from studies of preterm infants might not provide a comprehensive picture of NEC in term infants, and further studies are needed. NEC is a critical neonatal intestinal disease with a multifactorial etiology and may result in multisystem organ failure^22^,^23^. Therefore, the identification of various complications of NEC, including peritonitis, respiratory failure, shock and other critical illnesses, in the present study was not surprising. Similar to previous studies, the most commonly identified complication in this study was sepsis^17^. A higher rate of peritonitis was observed in infants with EO-NEC than in those with LO-NEC. This difference was most likely due to the fact that stage III NEC was more frequently identified in the EO group than in the LO group. Therefore, further research studies focusing on EO-NEC pathogenesis should be conducted.

Prior to this study, only 4 studies had compared EO-NEC and LO-NEC infants^9^,^18^,^24^,^25^. The mortality rate in LO-NEC infants has been found to be higher^9^,^24^ or lower^25^ than that observed in EO-NEC infants in prior pilot studies (n = 39, 37, and 62 in those 3 studies, respectively). However, no significant differences in mortality were identified between LO-NEC and EO-NEC infants in the large sample multicenter study (n = 858) conducted by Yee *et al*.^18^. Of the aforementioned 4 studies, only the study conducted by Short^9^ focused on full-term infants, and the results of that study suggested that LO-NEC was associated with increased mortality in full-term infants. However, no differences in the rate of mortality were identified between EO-NEC and LO-NEC infants (16% vs. 11.7%, *P* = 0.331) in our study. These inconsistent findings may be explained, at least to some extent, by the presence of statistical bias due to the small sample size (n = 39, 7 died) included in the study conducted by Short^9^. Thus, because we included a larger sample of patients, the results of our study may more comprehensively reflect the effects of NEC in full-term infants.

Many risk factors, including sepsis, respiratory failure, stage III NEC, intestinal perforation and peritonitis, have previously been reported to be associated with poor outcomes in infants with NEC^10^,^26^,^27^,^28^,^29^, most of which were also associated with poor prognosis in full-term infants with NEC in the present study. Additionally, patients in the nonsurvivor group more frequently required dopamine support and plasma transfusion relative to survivor group patients. Respiratory failure, peritonitis and kidney failure were identified as independent risk factors for poor diagnosis in the multivariate logistic regression analysis. Respiratory failure and peritonitis have been reported as risk factors for poor prognosis in previous studies^10^,^26^,^28^. Kidney failure, one of the most severe complications of NEC^30^, was first identified as a risk factor for NEC mortality in a pilot study (n = 19) conducted by Rivera-Moreno in 1999 31. The findings of the present study further demonstrated the association between kidney failure and mortality, and further studies should be conducted to clarify the molecular mechanism underlying this association. Short^9^ found that LO-NEC patients with lethal congenital heart disease (pulmonary atresia, single ventricle) were at increased risk of mortality. No cases of complex congenital heart disease were identified in the present study, and significant differences in the rates of common congenital heart diseases (ASD, VSD, PDA) were not identified between two groups, which may suggest that these common heart defects did not affect prognosis in our NEC patient population.

The results of the subgroup analyses indicated that peritonitis and kidney failure were independent risk factors for mortality in infants with EO-NEC and that peritonitis and respiratory failure were independent risk factors for morality in LO-NEC infants. While the exact cause of this etiological difference is unclear, six of the 7 infants with renal failure had EO-NEC and 4 had stage III NEC. Thus, we speculate that possible reasons for this difference are as follows: (1) fasting and weight loss and the development of serious illnesses during the first 7 days of life might lead to acute renal failure; and (2) the median age at EO-NEC onset was 2 days, which might result in medical staff having inadequate time to prevent the deterioration of NEC patients. Among infants with LO-NEC, high levels of endotoxins and proinflammatory cytokines may lead to multiple organ failure, including respiratory failure^22^,^23^, thereby potentially causing hypoxia of the intestinal tissue if respiratory failure results in damage to the intestinal epithelial cells. Thus, respiratory failure, which may occur as a result of NEC progression, may also be associated increased NEC mortality.

Limitations to our study include the errors and bias inherent to the retrospective nature of the study. Additionally, some patients were transferred to our center from other hospitals, and data regarding the details of treatment protocols performed outside of our hospital were limited. Furthermore, our investigations of the relationship between enteral feeding and NEC onset were limited due to a lack of data regarding the enteral feeding practices utilized outside of the hospital setting (such as the exact timing of feeding initiation and feeding quantity), which have been identified as important parameters in the assessment of NEC onset in full-term infants^6^,^11^,^32^,^33^. Therefore, further prospective studies should be conducted to clarify the mechanisms underlying NEC onset in full-term infants.

In general, the results of our study suggested that the median ages at NEC onset were 2 days among EO-NEC infants and 14 days among LO-NEC infants. The characteristics of the severe medical conditions identified in infants with EO-NEC were distinct from those observed in infants with LO-NEC. However, similar rates of mortality were observed in infants with early- or late-onset NEC. Peritonitis and kidney failure were found to be associated with mortality in infants with EO-NEC, while peritonitis and respiratory failure were significant predictors of mortality in LO-NEC infants.

## Additional Information

**How to cite this article:** Li, Q.-Y. *et al*. Differences in the Clinical Characteristics of Early- and Late-Onset Necrotizing Enterocolitis in Full-Term Infants: A Retrospective Case-Control Study. *Sci. Rep.* **7**, 43042; doi: 10.1038/srep43042 (2017).

**Publisher's note:** Springer Nature remains neutral with regard to jurisdictional claims in published maps and institutional affiliations.

## Figures and Tables

**Table 1:** Baseline characteristics of NEC patients.

Variables	Total (n = 253)	Early onset (n = 150)	Late onsetn (n = 103)	Statistics	*P*
	%(n), Mean ± S.D, M(P_25_-P_75_)				
Male	60.9 (154)	67.5 (98)	54.8 (56)	*χ*^*2*^ = 3.082	0.079
GA, week	39.04 ± 1.16	39.2 ± 1.19	38.82 ± 1.11	*t* = 2.541	0.012
Multiple gestation	3.6 (9)	2 (3)	5.8 (6)	*χ*^*2*^ = 1.609	0.205
Birth weight, kg	3.11 ± 0.51	3.16 ± 0.49	3.04 ± 0.53	*t* = 1.748	0.082
Small for gestation age	22.1 (56)	22 (33)	22.3 (23)	*χ*^*2*^ = 0.004	0.95
Vaginal delivery	39.9 (101)	45.3 (68)	34.0 (33)	*χ*^*2*^ = 3.854	0.05
Asphyxia	4.3 (11)	4.7 (7)	3.9 (4)	*χ*^*2*^ = 0.000	1
PROM (≥18 h)	2.8 (7)	2.7 (4)	2.9 (3)	*χ*^*2*^ = 0.000	1
Amniotic fluid contamination	12.65 (32)	14 (21)	10.7 (11)	*χ*^*2*^ = 0. 609	0.435
PIH	2.8 (7)	2.7 (4)	2.9 (3)	*χ*^*2*^ = 0.000	1
IDM	3.6 (9)	4 (6)	2.9 (3)	*χ*^*2*^ = 0.013	0.91
ICP	1.6 (4)	1.3 (2)	1.9 (2)	*χ*^*2*^ = 0.000	1
Feeding					
No feeding	7.1 (18)	8.7 (13)	4.9 (5)	*χ*^*2*^ = 3.44	0.179
Breast feeding	22.1 (56)	18.7 (28)	27.2 (28)		
Formula feeding	70.8 (179)	72.7 (109)	68 (70)		
Age at onset, d	5.67 (1–12)	2 (0.85–4)	14 (10–20)	*Z* = 13.523	0.000
Age at diagnosis, d	8 (4.07–14.94)	5 (3–7.43)	17 (11.38–24)	*Z* = 12.946	0.000
NEC stage					
Stage II	78.7 (199)	72.7 (109)	87.4 (90)	*χ*^*2*^ = 7.873	0.005
Stage III	21.3 (54)	27.3 (41)	12.6 (13)		
Surgical intervention	81.5 (44/54)	82.9 (34/41)	76.9 (10/13)	*χ*^*2*^ = 0.006	0.94

GA: Gestational age; PROM: prolonged rupture of the membranes (>18 h); PIH: pregnancy-induced hypertension; ICP: intrahepatic cholestasis of pregnancy; IDM: infants of diabetic mothers.

**Table 2:** Risk factors associated with prognosis in NEC infants.

Variables	Survivor group (n = 217)	Nonsurvivor group (n = 36)	Statistics	*P*
Respiratory failure, % (n)	2.3 (5)	27.8 (10)	*χ*^*2*^ = 31.5	0.000
Shock, % (n)	0.9 (2)	16.7 (6)	*χ*^*2*^ = 20.12	0.000
Sepsis, % (n)	21.7 (47)	63.9 (23)	*χ*^*2*^ = 27.515	0.000
Peritonitis, % (n)	8.3 (18)	61.1 (22)	*χ*^*2*^ = 64.711	0.000
Kidney failure, % (n)	0.9 (2)	13.9 (5)	*χ*^*2*^ = 14.78	0.000
Stage III NEC, % (n)	13.8 (30)	66.7 (24)	*χ*^*2*^ = 51.356	0.000
MODS, % (n)	0	5.6 (2)		0.02^*^
Heart failure, % (n)	0	5.6 (2)		0.02^*^
Required dopamine support, % (n)	15.2 (33)	30.6 (11)	*χ*^*2*^ = 5.063	0.024
Required plasma support, % (n)	10.6 (23)	30.6 (11)	*χ*^*2*^ = 8.925	0.003

MODS: Multiple organ dysfunction syndrome; *p-value for Fisher’s exact test.

**Table 3:** Risk factors associated with prognosis in early- and late-onset NEC infants [%(n)].

Variables	Early-onset group (n = 150)				Late-onset group (n = 103)			
	Survivor group (n = 126)	Nonsurvivor group (n = 24)	*χ*^*2*^	*P*	Survivor group (n = 91)	Nonsurvivor group (n = 12)	*χ*^*2*^	*P*
NEC stage III,% (n)	18.3 (23)	75 (18)	32.684	0.00	7.7 (7)	50 (6)	13.585	0.000^*^
Complications after NEC								
Peritonitis, % (n)	11.9 (15)	66.7 (16)	33.61^*^	0.00	3.3 (3)	50 (6)	23.438	0.000^*^
Sepsis, % (n)	24.6 (31)	66.7 (16)	16.579	0.00	16.7 (16)	58.3 (7)	7.938	0.005
Respiratory failure, % (n)	2.4 (3)	25 (6)	14.497^*^	0.00	2.2 (2)	33.3 (4)		0.001^*^
Kidney failure	1.6 (2)	16.7 (4)		0.006^*^	0	8.3 (1)		0.117^*^
Shock, % (n)	1.6 (2)	16.7 (4)		0.006^*^	0	16.7 (2)		0.013^*^
MODS, % (n)	0	8.3 (2)		0.025^*^	0	0		
Heart failure, % (n)	0	4.2 (1)		0.16^*^	0	8.3 (1)		0.117^*^

MODS: Multiple organ dysfunction syndrome; *p-value for Fisher’s Exact Test.

**Table 4:** Independent risk factors associated with prognosis in NEC infants.

Variables	*β*	*SE*	*Wald*	*P*	OR	95%CI
Total						
Respiratory failure	1.886	0.732	6.634	0.01	6.595	1.57–27.71
Kidney failure	2.614	1.048	6.224	0.013	13.656	1.752–106.466
Peritonitis	2.672	0.455	34.461	0.000	14.465	5.928–35.297
Early-onset group						
Kidney failure	3.016	1.032	8.541	0.003	10.331	2.7–154.173
Peritonitis	2.862	0.555	26.563	0.000	17.49	5.891–51.931
Late-onset group						
Respiratory failure	2.487	1.125	4.888	0.027	12.03	1.326–109.141
Peritonitis	3.024	0.886	11.65	0.001	20.579	3.624–116.85
